# Development of a Bamlanivimab Infusion Process in the Emergency Department for Outpatient COVID-19 Patients

**DOI:** 10.3390/healthcare10010042

**Published:** 2021-12-27

**Authors:** Danny H. Pham, Sandy Wong, Christina T. Nguyen, Stephen C. Lee, Kimberly J. Won

**Affiliations:** 1 University of California Irvine Medical Center, Orange, CA 93868, USA; dannyhp@hs.uci.edu (D.H.P.); stephcl2@uci.edu (S.C.L.); 2 School of Pharmacy, Chapman University, Irvine, CA 92618, USA; sanwong@chapman.edu (S.W.); christnguyen@chapman.edu (C.T.N.); 3 Providence Mission Hospital, Mission Viejo, CA 92691, USA

**Keywords:** coronavirus, bamlanivimab, emergency department infusion center

## Abstract

The coronavirus disease 2019 (COVID-19) pandemic has prompted the creation of new therapies to help fight against the novel severe acute respiratory syndrome coronavirus 2 (SARS-CoV-2). Bamlanivimab is a SARS-CoV-2 monoclonal antibody that is administered as an intravenous infusion to ambulatory patients with mild or moderate COVID-19, but a concern that arose was deciding the optimal location for patients to receive the medication. This report describes the development and implementation of a bamlanivimab infusion center in the emergency department of three hospitals in Orange County, California, shortly after bamlanivimab received emergency use authorization. As a result, a total of 601 patients received bamlanivimab in one of these three emergency departments between December 2020 to April 2021. The emergency department was shown to be an optimal setting for administration of bamlanivimab due to its convenience, accessibility, and capabilities for monitoring patients.

## 1. Introduction

The coronavirus disease 2019 (COVID-19) pandemic has resulted in over 247 million cases of infection and over 5 million deaths [1]. This public health crisis has encouraged prompt responses to discover new agents and therapies that may prevent or treat COVID-19 caused by the novel severe acute respiratory syndrome coronavirus 2 (SARS-CoV-2). One new treatment involves administering anti-SARS-CoV-2 antibodies to potentially reduce viral load, improve symptoms, and prevent hospitalization [2,3]. In November 2020, bamlanivimab (LY-CoV555) was the first monoclonal antibody to be issued an emergency use authorization (EUA) by the U.S. Food and Drug Administration (FDA) for the treatment of mild-to-moderate COVID-19 in non-hospitalized adult and pediatric patients at high risk for progressing to severe disease and/or hospitalization [4]. Bamlanivimab is a recombinant human immune globulin G kappa (IgG1κ) monoclonal antibody that is derived from convalescent plasma [4]. It neutralizes the surface spike glycoprotein of SARS-CoV-2, preventing spike protein attachment to the human angiotensin-converting enzyme 2 (ACE2) receptor and preventing entry of the virus into human cells [5]. Bamlanivimab is administered as a one-time intravenous (IV) infusion over ≥60 min [4]. The phase 2 BLAZE-1 trial (n = 452) reported that bamlanivimab, given at a dose of 2800 mg, significantly reduced the viral load at day 11 (−0.53 difference 95% confidence interval (CI), −0.98 to −0.08; *p* = 0.02) compared to placebo. Additionally, COVID-19-related hospitalizations at day 29 were 1.6% in the bamlanivimab group vs. 6.3% in the placebo group [6].

Both the National Institute of Health and Infectious Diseases Society of America recommend the use of neutralizing antibodies for qualifying patients [7,8]. As bamlanivimab is authorized for use in non-hospitalized patients, there are logistical concerns with how patients would receive this IV medication safely. Given the potential antigenicity and lack of long-term data with monoclonal antibodies with EUA for COVID-19, bamlanivimab should be infused in settings where health care providers who have the knowledge and experience to treat severe infusion reactions can monitor patients for adverse effects and infusion-related reactions for at least one hour post-infusion [4]. Traditional outpatient infusion centers are options for administering the infusion due to the established infrastructure and resources, but there is concern regarding exposing high-risk patients (i.e., immunocompromised) who commonly frequent these centers to COVID-19 positive individuals. Furthermore, at the beginning of the pandemic, these centers were actually identified as potential sources of SARS-CoV-2 transmission, and other options were needed to decrease this risk [9]. Additionally, an uptake of administering COVID-19 antibody treatment could also cause an infusion center to become impacted.

The emergency department (ED) provides a unique setting for the implementation of a monoclonal antibody infusion site for outpatient treatment. The benefits for using an ED to infuse monoclonal antibodies are (1) more resources for medication preparation and administration, (2) the ability to isolate patients, and (3) close monitoring by emergency medicine-trained personnel. Additionally, patients presenting to the ED with COVID-19 symptoms could be immediately screened for eligibility and receive the monoclonal antibodies during the same visit. Institutions, like George Washington University and Mount Sinai Medical Center, have reported successful implementation of ED administration of COVID-19 antibody therapies [10,11].

In this paper, we describe the workflow and impact of administering IV monoclonal antibodies in the ED of two large healthcare systems.

## 2. Materials and Methods

The implementation of a COVID-19 monoclonal antibody infusion program in the ED was established at three hospitals in California: Providence Mission Hospital Mission Viejo (Mission Viejo, CA, USA), Providence Mission Hospital Laguna Beach (Laguna Beach, CA, USA), and the University of California, Irvine Medical Center (Orange, CA, USA).

### 2.1. Providence Mission Hospital Mission Viejo and Providence Mission Hospital Laguna Beach

**Figure 1:**
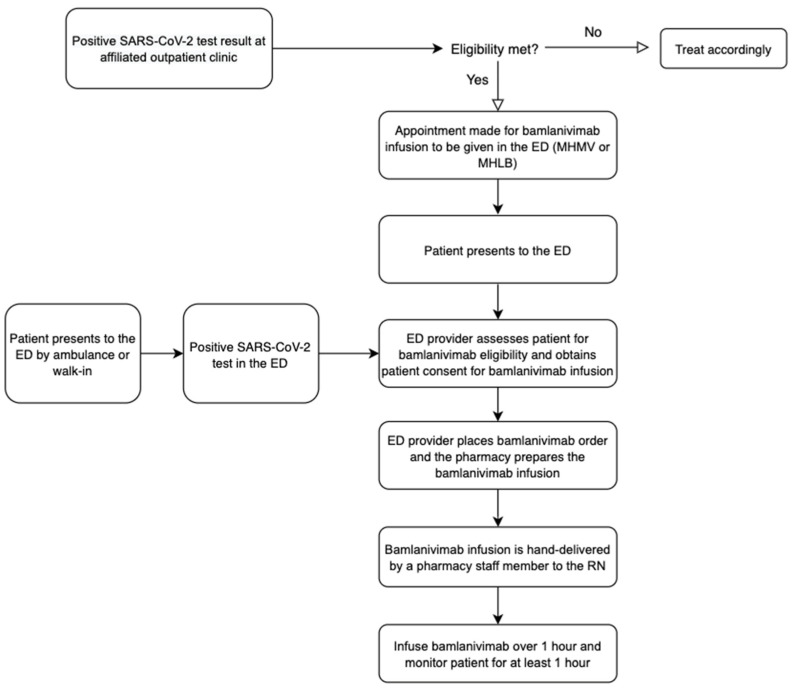
Workflow for bamlanivimab infusion in the ED of Providence MHMV and MHLB.

Providence Mission Hospital Mission Viejo (MHMV) is a 504-bed acute care hospital in Mission Viejo, California. Providence Mission Hospital Laguna Beach (MHLB) is a 189-bed acute care hospital in Laguna Beach, California. Patients with mild-to-moderate COVID-19 symptoms could go about receiving bamlanivimab in the ED through one of two processes: (1) by referral from an affiliated outpatient clinic or (2) by presenting directly to the ED, either by walk-in or by ambulance. (Figure 1). Regardless of the setting, all patients were screened using the bamlanivimab EUA inclusion criteria provided by the FDA. (Table 1).

If a patient presented to any affiliated outpatient clinic and was diagnosed with mild-to-moderate COVID-19 not requiring hospitalization, the patient would be referred to the nursing coordinator in charge of scheduling COVID-19 antibody infusion appointments. The nursing coordinator would determine the patient’s eligibility for receiving bamlanivimab and then schedule an appointment for the patient to receive the monoclonal antibody infusion in the ED at MHMV or MHLB, based on the patient’s preference. Initially, only two patients per day were scheduled between 1200–1900, 7 days a week. The patient would be instructed to present at their scheduled appointment time to the “tent” near the entrance of the ED, where they would check in with the intake nurse. Conversely, patients who presented directly to the ED to be evaluated for COVID-19-related symptoms would be brought back to an isolated ED room or placed in the Respiratory Waiting Room if there were no ED rooms available.

Regardless of if the patient was referred from the outpatient clinic or presented directly to the ED with COVID-19 symptoms, they were re-evaluated by an ED provider (e.g., physician, physician assistant, or nurse practitioner). If eligible for monoclonal antibody administration, the ED provider would provide the patient with a copy of the EUA fact sheet, get consent to give bamlanivimab, document the patient’s eligibility in their progress note, and then place an order for the bamlanivimab infusion in the patient’s electronic health record (EHR). The bamlanivimab infusion was a standalone order (i.e., not a part of an order set). Once the order was placed, the pharmacist would confirm the patient’s eligibility, verify the order, and then print a label for the pharmacy technician to begin preparing the medication in the IV room. Two bamlanivimab infusions were premade in the morning to cover the two scheduled appointments. Any additional bamlanivimab orders received outside of the two scheduled appointments were compounded once the label printed. After the bamlanivimab was prepared, a pharmacy technician would hand-deliver the medication to the ED nurse who was assigned to administer the antibody infusions for that shift (the “Rapid Assessment Nurse”). 

Patients who were referred and had a scheduled appointment would receive the infusion in the tent outside next to the ED, whereas those who presented directly to the ED would receive their infusion wherever there was an available room—either in an assigned ED room, the tent outside next to the ED, or if there were less than four infusions and the patient presented outside of the tent operation times (1200–2330), the patient might need to receive the infusion in the ED respiratory waiting room. Vitals and symptoms were recorded prior to, during and after the infusion. Patients were observed for at least one hour post-infusion. Supportive medications for any adverse effects were ordered as needed at the provider’s discretion. If no complications arose, the patient would be discharged from the ED and asked to follow up with their primary care physician in 2 days.

A list of patients who received a bamlanivimab infusion was kept on the hospital’s secure server so that a nurse dedicated to conducting ED follow-up telephone calls would be able to call the patient. Patients would receive a follow-up phone call within 1–5 days of receiving their bamlanivimab infusion. During this telephone encounter, the nurse would ask the patient questions related to the their COVID-19 symptoms, as well as questions to determine any potential allergic reactions or adverse effects to the bamlanivimab. All information provided during this telephone encounter was documented on a spreadsheet for internal use.

### 2.2. University of California Irvine Medical Center 

University of California Irvine Medical Center (UCIMC) is a 417-bed acute care hospital in Orange, California. When a patient presents to the ED with COVID-19 symptoms, they are screened by the ED physician or advanced practice provider for severity of infection and eligibility of receiving bamlanivimab infusion based on the inclusion and exclusion criteria in the FDA EUA (Table 1). If eligible, they would be admitted to a designated area in the ED to receive the medication. Priority for monoclonal antibody administration was based on a point system, where patients were given a range of 1 to 4 points depending on their age, gender, and concurrent comorbidities. Patients with the highest points were prioritized to receive bamlanivimab (Table 2). 

**Figure 2:**
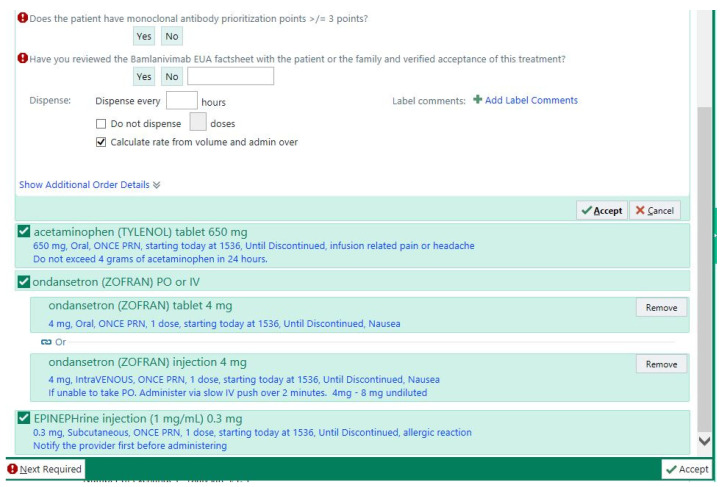
Bamlanivimab order set. The bamlanivimab order set contained two required questions that the pharmacist had to answer, as well as supportive medications in case they were needed.

**Figure 3:**
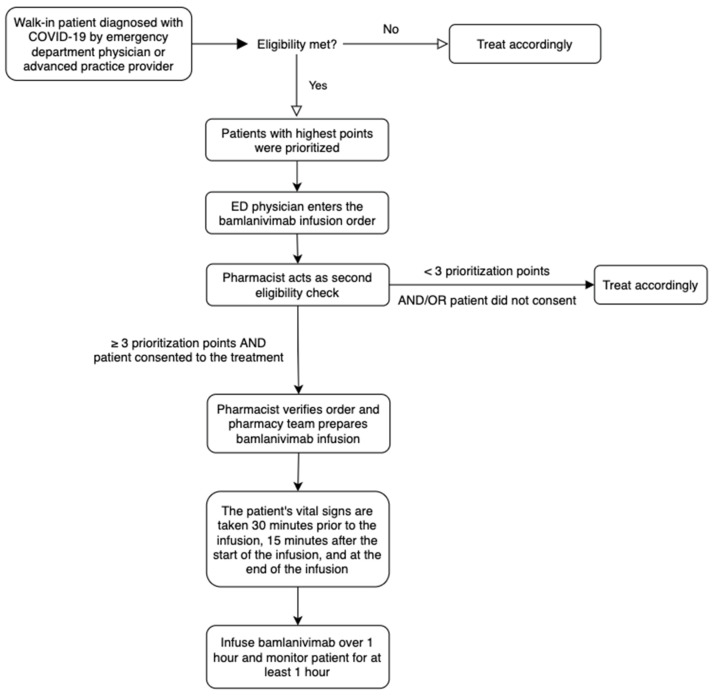
Workflow for bamlanivimab infusion in the ED of UC Irvine Medical Center.

After the ED physician reviewed the EUA fact sheet with the patient and received consent to give the medication, the physician would input the order set into the EHR and the order would be verified by a pharmacist. To ensure that the patient was eligible and met ≥ 3 prioritization points (Table 2), the pharmacist was required to answer two questions related to the patient’s eligibility, which were embedded into the EHR’s order set (Figure 2). The bamlanivimab order set included the monoclonal antibody, along with as needed orders for supportive medications, such as acetaminophen, ondansetron, and epinephrine in case of an anaphylactic reaction (Figure 3).

After the bamlanivimab order set was verified, the inpatient pharmacy team would prepare the medication. Once completed, the medication would be delivered to the unit. The infusion nurse would follow the bamlanivimab administration protocol for proper administration. Vital signs were taken at three different time points: within 30 min of starting the infusion, 15 min after the start of infusion, and at the end of the infusion. After the bamlanivimab was administered, the patient would be observed for at least 1 h for hypersensitivity/infusion-related reactions. If no complications arose, the patient would be discharged from the ED (Figure 3).

## 3. Results

The bamlanivimab infusion workflow was implemented on 20 December 2020 at Providence MHMV and MHLB locations. A total of 341 patients received bamlanivimab infusion from 20 December 2020 to 20 March 2021. At UCIMC, the bamlanivimab infusion workflow was implemented on 3 December 2020 and the first patient was treated on 4 December 2020. From 4 December 2020 to 15 April 2021, a total of 260 patients received a bamlanivimab infusion. Collectively between the two health systems, 601 patients received a bamlanivimab infusion. The mean age was 62 ± 16 years and about half were male (n = 308, 51.2%). The most common high-risk eligibility criteria identified were hypertension (56.6%), followed by diabetes (28.3%), immunosuppressive disease or on immunosuppressive therapy (23.6%), and heart disease (23.5%) (Table 3). 

Of the 601 patients who received a bamlanivimab infusion, 87 patients (14.5%) returned to the hospital for COVID-19 symptoms within 10 days of bamlanivimab administration. Of these patients, 43 (7.2%) were admitted for COVID-19 infection (Table 4).

## 4. Discussion

A total of 601 patients received bamlanivimab infusion in the ED for mild-to-moderate COVID-19 during the study period. Of the eighty-seven patients (14.5%) patients who returned to the hospital for COVID-19-related illness, only 43 (7.2%) were admitted for COVID-19 infection, resulting in a 92.8% hospitalization avoidance. However, these results should be considered with caution as they only include return visits and admissions that occurred at one of the three study institutions. Additionally, given the retrospective design of this report, there are limitations with the breadth of data collection. Nonetheless, our findings are similar to other prospective studies that have reported significant reductions in ED visits or hospitalizations with bamlanivimab monotherapy as a secondary outcome [6,12,13].

Despite the successful implementation of a bamlanivimab infusion program in the ED, administration of bamlanivimab monotherapy was discontinued in March/April 2021 due to the rise of resistant strains of COVID-19 to bamlanivimab. On 19 March 2021, the California Department of Public Health recommended facilities and providers to stop administering bamlanivimab monotherapy in California due to the rise of B.1.429/B.1.427 SARS-CoV2 variants [14]. Furthermore, on 16 April 2021, the FDA revoked bamlanivimab’s EUA due to the updated BLAZE-1 phase 2 study results, which showed reduced efficacy of bamlanivimab monotherapy compared to placebo [12,13,15]. Consequently, both institutions sought an alternative treatment option to replace bamlanivimab monotherapy.

Casirivimab and imdevimab are two recombinant human monoclonal antibodies that bind to non-overlapping epitopes of the SARS-CoV-2 spike protein receptor binding domain to inhibit the binding to the ACE2 receptor, thus, preventing entry of the virus [16]. The combination of casirivimab and imdevimab (REGN-COV2) was given EUA by the FDA on 21 November 2020 based on the results from the R10933-10987-COV-2067 trial [17]. This study was a randomized, double-blind, placebo-controlled clinical trial conducted in ambulatory adults with mild-to-moderate COVID-19 symptoms (n = 799) who were treated with a single infusion of 2400 mg (1200 mg casirivimab/1200 mg imdevimab), 8000 mg (4000 mg casirivimab/4000 mg imdevimab), or placebo [17]. As a result, both institutions switched from using bamlanivimab to REGN-COV2. The same infusion protocol was kept even after the medication switch since the ED proved to be an optimal location for other similar infusion processes.

The combination of bamlanivimab and etesevimab is also another monoclonal antibody combination that was granted EUA by the FDA based on the updated results of the BLAZE-1 study [18]. In phase 3 of the BLAZE-1 trial, ambulatory patients with mild-to-moderate COVID-19 symptoms (n = 1035) who were treated with a single infusion of 2800 mg of bamlanivimab and 2800 mg of etesevimab (n = 518) had a lower incidence of COVID-19-related hospitalization and death compared to placebo (n = 517) [19]. The combination of bamlanivimab and etesevimab also showed a greater reduction and accelerated the decline in SARS-CoV-2 viral load compared to placebo [19].

A concern with the implementation of the infusion program in the ED is the strain it could have on the department as a patient receiving antibodies takes up space and staffing resources that could be used for other acute care patients. The American College of Emergency Physicians published a statement supporting monoclonal antibody infusions in the ED as long as hospitals can ensure that there is adequate resources to care for emergency patients [20]. At UCIMC, we did not notice any strains on the department as the process was streamlined and the patients did not require intensive care. Furthermore, extra space was added to help mediate a potential impacted ED. For example, the implementation of outdoor mobile tents to triage patients in the ED helped create more room. On the other hand, at Providence MHMV, though there was already a tent set up outside the ED for isolation of potential COVID-19 patients, the ED nursing and pharmacy staff experienced a strain with the additional time consumption and use of resources used for the implementation of an infusion program. To work through these challenges, administrators from both departments and key stakeholders should work together to develop a workflow that works for the institution. For example, the maximum of two monoclonal antibody infusion appointments per day was initiated post-implementation of the infusion program to address concerns of the staff involved.

Outpatient infusion centers could be utilized as an additional site of monoclonal antibody administration to alleviate strain in the ED, but many patients who frequent the outpatient infusion center are immunocompromised and could be put at higher risk if they come in contact with patients diagnosed with COVID-19. Additionally, scheduling patients is a major concern with infusion centers, and patients requiring monoclonal antibody administration could have delay of care because of this. Many patients also presented to the ED directly with a diagnosis of mild-to-moderate COVID-19 and referring them to an outpatient infusion center could also cause a delay of care.

## 5. Conclusions

This paper describes the successful implementation of two different bamlanivimab infusion programs in the EDs of two large health systems. Despite having to rapidly develop the workflows in a short period of time after the bamlanivimab EUA was approved, there were no major workflow issues that occurred, regardless of whether patients presented directly to the ED or were referred/scheduled for a bamlanivimab appointment. Given the vulnerability of patients in infusion centers and the logistical and safety considerations of monoclonal antibody IV administration, the ED is an optimal setting due to its patient accessibility, capacity to isolate and monitor patients, and emergency medicine-trained personnel who have the expertise to manage severe allergic and infusion-related reactions.

The methods described in this paper could be utilized for future administrations of monoclonal antibodies/infusions for COVID-19 or other disease states in the ED.

## Figures and Tables

**Table 1:** Bamlanivimab EUA Inclusion and Exclusion Criteria. The original bamlanivimab EUA criteria only included adult patients (i.e., age ≥ 18 years old). In February 2021, the FDA updated the inclusion criteria to include pediatric patients ≥12 years old.

**Inclusion Criteria:** Adult ^‡^ and pediatric patients (12 years of age and older weighing at least 40 kg) with positive results of direct SARS-CoV-2 viral testing and who are at high risk for progressing to severe COVID-19 and/or hospitalization. High risk is defined as patients who meet at least one of the following criteria: ^‡^ Have a body mass index (BMI) ≥ 35 kg/m^2^; ^‡^ Have chronic kidney disease; ^‡^ Have diabetes; ^‡^ Have immunosuppressive disease; ^‡^ Are currently receiving immunosuppressive treatment; ^‡^ Are ≥ 65 years of age; ^‡^ Are ≥ 55 years of age AND have: ○ Cardiovascular disease, OR ○ Hypertension, OR ○ Chronic obstructive pulmonary disease/other chronic respiratory disease. Are 12–17 years of age AND have: ○ BMI ≥ 85th percentile for their age and gender based on CDC growth charts, https://www.cdc.gov/growthcharts/clinical_charts.htm (accessed on 10 November, 2021).; OR ○ Sickle cell disease; OR ○ Congenital or acquired heart disease; OR ○ Neurodevelopmental disorders, for example, cerebral palsy; OR ○ A medical-related technological dependence, for example, tracheostomy, gastrostomy, or positive pressure ventilation (not related to COVID-19); OR ○ Asthma, reactive airway, or other chronic respiratory disease that requires daily medication for control.
**Exclusion Criteria** Patients who: ○ Are hospitalized due to COVID-19; ○ Require oxygen therapy due to COVID-19; ○ Require an increase in baseline oxygen flow rate due to COVID-19 in those on chronic oxygen therapy due to underlying non-COVID-19 related comorbidity; ○ Received bamlanivimab through clinical trial or compassionate use, or from another EUA supply; ○ Has a contraindication to bamlanivimab (known hypersensitivity).

^‡^ Denotes original EUA criteria approved in November 2020.

**Table 2:** Prioritization points for bamlanivimab administration. Priority for monoclonal antibody administration was based on a point system, where patients were given a range of 1 to 4 points depending on their age, gender, and concurrent comorbidities.

Age ≥ 80 years (4 points)
Age 65–79 years (2 points)
Age ≥ 50 years (1 point)
Solid organ transplant (3 points)
Body mass index (BMI) ≥ 35 kg/m^2^ (2 points)
Hematologic cancer (2 points)
Male sex (1 point)
Ethnicity non-white (1 point)
Chronic kidney disease (1 point)
Cirrhosis (1 point)
COPD (1 point)
Hypertension (1 point)
Heart disease (1 point)
Diabetes (1 point)
Recent (within 1 year) non-hematologic cancer (1 point)
Currently receiving immunosuppressive treatment (1 point)
Pregnancy (1 point)
Symptoms < 4 days (1 point)

**Table 3:** Patient demographics (n = 601).

Age, years (Mean ± SD)	62 ± 16
Male, n (%)	308 (51.2)
Hypertension, n (%)	340 (56.6)
Chronic kidney disease, n (%)	79 (13.1)
Immunosuppressive disease or on immunosuppressive treatment, n (%)	142 (23.6)
Body mass index ≥ 35 kg/m^2^, n (%)	109 (18.1)
Diabetes, n (%)	170 (28.3)
Asthma, n (%)	98 (16.3)
Chronic obstructive pulmonary disorder, n (%)	25 (4.2)
Sickle cell anemia, n (%)	9 (1.5)
Heart disease (congenital or acquired), n (%)	141 (23.5)
Neurodevelopmental disorders, n (%)	101 (16.8)
Medical-related device dependence *, n (%)	16 (2.7)

* including tracheostomy, gastronomy, positive pressure ventilation, etc.

**Table 4:** Patient results (n = 601).

Hospitalization for COVID-19 within 10 days of receiving bamlanivimab, n (%)	87 (14.5)
Admission for COVID-19 within 10 days of receiving bamlanivimab, n (%)	43 (7.2)

## Data Availability

Data available on request from the authors.

## References

[1] WHO Coronavirus (COVID-19) Dashboard World Health Organization. https://covid19.who.int/.

[2] Ju B., Zhang Q., Ge J., Wang R., Sun J., Ge X. (2020). Human neutralizing antibodies elicited by SARS-COV-2 infection. Nature.

[3] Taylor P.C., Adams A.C., Hufford M.M., de la Torre I., Winthrop K., Gottlieb R.L. (2021). Neutralizing monoclonal antibodies for treatment of COVID-19. Nat. Rev. Immunol..

[4] Fact Sheet for Health Care Providers Emergency Use Authorization (eua) of Bamlanivimab. https://www.fda.gov/media/143603/download.

[5] ACTIV-3/TICO LY-CoV555 Study Group (2020). A neutralizing monoclonal antibody for hospitalized patients with COVID-19. N. Engl. J. Med..

[6] Chen P., Nirula A., Heller B., Gottlieb R.L., Boscia J., Morris J., Huhn G. (2021). SARS-COV-2 neutralizing antibody LY-Cov555 in outpatients with Covid-19. N. Engl. J. Med..

[7] COVID-19 Treatment Guidelines Panel Coronavirus Disease 2019 (COVID-19) Treatment Guidelines. https://www.covid19treatmentguidelines.nih.gov/.

[8] Bhimraj A., Morgan R.L., Shumaker A.H. Infectious Diseases Society of America Guidelines on the Treatment and Management of Patients with COVID-19. https://www.idsociety.org/practice-guideline/covid-19-guideline-treatment-and-management/.

[9] Dotan I., Panaccione R., Kaplan G.G., O’Morain C., Lindsay J.O., Abreu M.T. (2020). Best Practice Guidance for Adult Infusion Centres during the COVID-19 Pandemic: Report from the COVID-19 International Organization for the Study of IBD [IOIBD] Task Force. J. Crohn’s Colitis.

[10] Payette C., Brooks J.T., Shesser R. (2021). Emergency department administration of COVID-19 antibody therapies: Early experience. Am. J. Emerg. Med..

[11] Jodoin K., Farcy D., Caldera A., Dalley M., Patel B., Stuart W., Telas G., Atisha M. (2021). Covid-19 monoclonal antibody infusions: A multidisciplinary initiative to operationalize EUA Novel Treatment Options. J. Clin. Outcomes Manag..

[12] Cohen M.S., Nirula A., Mulligan M.J. (2021). Effect of bamlanivimab vs placebo on incidence of COVID-19 among residents and staff of skilled nursing and assisted living facilities—A randomized clinical trial. JAMA.

[13] Gottlieb R.L., Nirula A., Chen P. (2021). Effect of Bamlanivimab as Monotherapy or in Combination with Etesevimab on Viral Load in Patients with Mild to Moderate COVID-19: A Randomized Clinical Trial. JAMA.

[14] California Department of Public Health (2021). Health Alert: Concerns re: The use of Bamlanivimab Monotherapy in the Setting of SARS-CoV2 Variants. http://publichealth.lacounty.gov/eprp/lahan/alerts/CAHANBamlanivimabandSARSCoV2Variants.pdf.

[15] U.S. Food and Drug Administration (2021). Coronavirus (COVID-19) Update: FDA Revokes Emergency Use Authorization for Monoclonal Antibody Bamlanivimab. https://www.fda.gov/news-events/press-announcements/coronavirus-covid-19-update-fda-revokes-emergency-use-authorization-monoclonal-antibody-bamlanivimab.

[16] Weinreich D.M., Sivapalasingam S., Norton T. (2021). Regn-COV2, a neutralizing antibody cocktail, in outpatients with covid-19. N. Engl. J. Med..

[17] U.S. Food and Drug Administration (2020). Coronavirus (COVID-19) Update: FDA Authorizes Monoclonal Antibodies for Treatment of COVID-19. https://www.fda.gov/news-events/press-announcements/coronavirus-covid-19-update-fda-authorizes-monoclonal-antibodies-treatment-covid-19.

[18] U.S. Food and Drug Administration (2021). FDA authorizes Bamlanivimab and Etesevimab Monoclonal Antibody Therapy for Post-Exposure Prophylaxis (Prevention) for COVID-19. https://www.fda.gov/drugs/drug-safety-and-availability/fda-authorizes-bamlanivimab-and-etesevimab-monoclonal-antibody-therapy-post-exposure-prophylaxis.

[19] Dougan M., Nirula A., Azizad M. (2021). Bamlanivimab plus Etesevimab in Mild or Moderate Covid-19. N. Engl. J. Med..

[20] (2021). American College of Emergency Physicians. ACEP Statement on Monoclonal Antibody Infusion. https://www.acep.org/corona/COVID-19-alert/covid-19-articles/acep-statement-on-monoclonal-antibody-infusion/.

